# Eating Competence, Food Consumption and Health Outcomes: An Overview

**DOI:** 10.3390/ijerph19084484

**Published:** 2022-04-08

**Authors:** Fabiana Lopes Nalon de Queiroz, António Raposo, Heesup Han, Martín Nader, Antonio Ariza-Montes, Renata Puppin Zandonadi

**Affiliations:** 1 Department of Nutrition, Faculty of Health Sciences, Campus Universitário Darcy Ribeiro, University of Brasília, Brasilia 70910-900, Brazil; fabinalon@hotmail.com (F.L.N.d.Q.); renatapz@unb.br (R.P.Z.); 2 CBIOS (Research Center for Biosciences and Health Technologies), Universidade Lusófona de Humanidades e Tecnologias, Campo Grande 376, 1749-024 Lisboa, Portugal; 3 College of Hospitality and Tourism Management, Sejong University, Seoul 05006, Korea; 4 Department of Psychological Studies, Universidad ICESI, Cali 76001, Colombia; mnader@icesi.edu.co; 5 Social Matters Research Group, Universidad Loyola Andalucía, C/Escritor Castilla Aguayo, 4, 14004 Córdoba, Spain; ariza@uloyola.es

**Keywords:** eating competence, health, eating behavior, nutrition

## Abstract

Eating Competence (EC) is one behavioral perspective of eating practices that has been associated with a healthy lifestyle. It emphasizes eating pleasure, self-regulation of eating, body weight satisfaction, and regular meal frequency that includes food variety without focusing on dietary guidelines. EC is composed of four components (Eating Attitude, Food Acceptance, Internal Regulation, and Contextual Skill), and its assessment is performed using the Eating Competence Satter Inventory (ecSI2.0™), developed and validated in English for an adult population. EC has been associated with diet quality and health indicators for various population groups and the development of skills that increase EC might be a strategy to improve nutritional health, and prevent obesity and other chronic diseases. In this sense, this study presents an overview of the background, concepts, features, and possible associations among EC, food consumption, and health outcomes. The high prevalence of diseases associated with food/nutrition draws attention to the necessity to broaden the view on food and its relationship with health and well-being, considering not only nutrients and food combinations but also the behavioral dimensions of eating practices. Healthy nutritional recommendations that take into account attitudes and behaviors are in accordance with the EC behavioral model. Studies on eating behavior emphasize the need to better understand attitudes towards food and eating in the general population using validated instruments. In this context, measuring EC and its association with health outcomes seems to be relevant to nutritional health. The complexity of food choices has been examined in social, behavioral, and biological sciences, representing a great challenge for applying unique and simple theoretical models. Multiple methods are required, as no single theory can fully explain food selection.

## 1. Introduction

Eating is not only a basic human need for survival and health, but also a global activity that involves both internal and external aspects of the individual [1]. The main purpose of eating is to sustain life [2]; nonetheless, human nutrition reflects a lifestyle and is modeled by the social, cultural, and economic scenarios that depend on how food is generated, distributed, acquired, sold, prepared/cooked, and consumed [3].

The relationship between food and health seems quite obvious; however, the adoption of healthy eating practices is not restricted to the scope of individual choices, as physical, economic, political, cultural, and social factors influence people’s diet positively or negatively [3,4]. Unhealthy eating can be modified by encouraging changes in the individual’s behavior [5]. In this sense, concerning individual choices, emerging behavioral approaches show promise in improving eating patterns [6].

One behavioral perspective that has been associated with a healthy lifestyle is Eating Competence (EC), based on The Satter Eating Competence Model (ecSatter), which emphasizes eating pleasure, eating self-regulation, body weight contentment, and a regular meal routine that includes a range of foods without a focus on dietary guidelines [7]. EC involves four fundamental components (eating attitudes, food acceptance, internal regulation of food intake, and management of eating context) and was developed for nutrition education and to characterize eating-related behavior [2,7]. Several studies show that EC is related to diet quality and health indicators for various population groups [8,9,10,11], associated with an increased consumption of fruits and vegetables (FV) [8], adherence to the Mediterranean diet [12], and greater skills in managing the food context [13]. These characteristics meet the Food and Agriculture Organization of the United Nations (FAO) and World Health Organization (WHO) criteria regarding recommendations for healthy eating, which state that the development of skills to make decisions for a healthy diet is part of nutrition education strategies, as it supports the individual in understanding the determining factors of eating practices and encourages the adoption of health-promoting behaviors [3]. Furthermore, studies revealed that individuals with higher EC tend to be more physically active [14], have lower cardiovascular risk [11,12], and have better sleep quality [15,16]. EC is not restricted to the individual scope but is associated with positive eating habits by parents when feeding school-age children [17] and preschool children [18]. Findings also reveal that EC is linked to psychological and behavioral aspects, such as greater satisfaction with body weight and lower frequency of behaviors associated with eating disorders [9,19,20].

Considering that EC is associated with health indicators in several population groups, developing skills that increase EC might be a strategy to improve diet quality and prevent obesity and other chronic diseases. In the present review, we aimed to provide an insight into the background, concept, features, and potential associations among EC, food consumption, and health outcomes.

## 2. Behavioral Approaches Applied to Eating

The science of nutrition has been developed based on identifying and isolating nutrients present in foods and their effects on the incidence of certain diseases. Yet, it has been progressively insufficient to elucidate the reasons that motivate food choices. Thus, the knowledge about the nutritional aspect of food does not seem to be the biggest influencer of food consumption [21,22]. Emotional and behavioral responses, whose construction depends on different internal and external experiences, affect how individuals feed [23,24]. In this sense, the understanding of food choices goes beyond the definition of biological and nutritional needs. It is known that food choices are not always conscious; they often happen automatically and habitually, associated with what is available, considering economic aspects and food accessibility [24].

To develop eating practices consistent with health recommendations, behavioral aspects have been explored and valued, seeking to understand how individuals eat, serving as tools to modify their diet. A systematic review that selected 16 articles comparing traditional interventions (caloric restriction and focus on weight loss) with behavioral interventions, noted that the behavioral-based interventions resulted in statistically significant improvements in eating disorder patterns [25]. Even without the prescription of a meal plan, or significant weight loss, individuals undergoing behavioral approaches showed improvements in terms of health as they did not increase their weight in the long term; did not have any worsening in blood pressure, blood glucose patterns, or blood cholesterol levels; and also presented a significant improvement in biomarkers [25]. A limiting aspect of studies with alternative approaches is the natural difficulty in defining what is an approach without a dietary prescription, as some studies included nutritional counseling without specifying how they were carried out.

One of the behavioral approaches is Intuitive Eating, a concept first introduced in 1995 by the American nutritionists Evelyn Tribole and Elyse Resch [26]. It is characterized by teaching individuals to become aware of their bodies, basing food choices on the physiological signs of hunger and satiety and not on the emotional responses that lead to inadequate food consumption [27]. The intuitive approach discourages the practice of diets as a possible source of behavior change and advocates three pillars: unconditional permission to eat; eating to meet physiological and non-emotional needs, and relying on internal hunger and satiety signals to establish what and how much to eat [28,29].

The ability to eat intuitively can be measured using the Intuitive Eating Scale, which assesses the individual’s ability to watch out for internal signs of hunger and satiety [27]. The intuitive eating model and its health-promoting benefits have been extensively studied [30]. Recent findings show that individuals with higher scores on the Intuitive Eating Scale are successful in maintaining a stable weight over time, even though they do not have a strong desire to change their weight [31], which seems to be healthier than repeated cycles of weight loss and regain [32,33]. Learning to eat intuitively is a challenge, mainly due to the premise of having unconditional permission to eat [34], which can represent a barrier to its application in different population groups.

Another behavioral proposal is Mindful Eating, which is based on eating with a focus on physical and emotional sensations aroused during eating, without judgment or criticism [35,36]. The state of attention is not restricted to the food choice, but it also means being aware of the present time, paying attention to the meal, including the biological signs of hunger and satiety, and the emotions and environmental stimuli that lead to eating automatically and whilst distracted [37]. Increased attention allows conscious changes in eating habits, helping to eliminate negative eating patterns that tend to be repeated automatically and unconsciously in people’s daily lives. Thus, mindfulness in eating promotes a satisfactory and healthy relationship with “what”, “how much”, “where”, and “how” to eat [37]. Mindfulness is an ability learned [35] through meditation and practical exercises that have been widely publicized in the media, showing benefits in reducing stress, anxiety, insomnia, and binge eating [38,39]. The Mindful Eating Questionnaire (MEQ), developed and validated in the United States, is a tool designed to measure mindfulness in eating has been used by various researchers in different countries [35].

Mindfulness in eating has been shown to be a negative predictor of eating disorders in college students and is inversely related to binge eating [40]. An integrative literature review on the influence of mindfulness eating on weight loss, weight return, and weight maintenance evaluated 12 articles, showing positive weight loss outcomes when mindfulness strategies were employed [41]. Mindful Eating has been shown to lessen the harmful behaviors associated with overweight and obesity, emphasizing the act of tasting food andeating slowly and only when hungry [42]. As a learned skill, it requires engagement and discipline from its practitioners. However, a recent study showed that, in mindfulness weight loss programs, only a third of participants successfully practiced mindfulness exercises regularly [43]. This might become a limiting factor for the popularization of such a practice.

A more encompassing model with a rigorous examination is the concept of Eating Competence (EC), based on The Satter eating competence model (ecSatter), introduced in 2007 by the American nutritionist Ellyn Satter. According to this approach, eating is a multi-faceted activity involving learned behaviors, social expectations, lifelong preferences, and attitudes and sentiments about food and eating [7]. The ecSatter is based on the fact that hunger and satiety signals in the organism, when properly perceived, are reliable and must be attended to, serving to guide food selection, providing energy balance, and leading to stable body weight [7]. Skills and resources support this internal process for regulating the food environment to provide reliable and regular diets [7].

As a biopsychosocial approach, EC is not concerned with nutrients, portion sizes, or food groups, but rather appreciating food and eating, paying attention to diet diversity, hunger, and satiety sensations, and often making meals while keeping nutrition and the food consumption environment in mind [2]. Such skills are encompassed in four components: Food Acceptance; Eating Attitude; Internal Regulation; and Contextual Skills [2,7]. Individuals who have a higher EC tend to deal better with food and eating, showing self-confidence in relation to food choices, as well as willingness and openness to new food experiences, achieving a balance between desires, choices, and quantities to be ingested [2].

## 3. Components of Eating Competence

**Figure 1:**
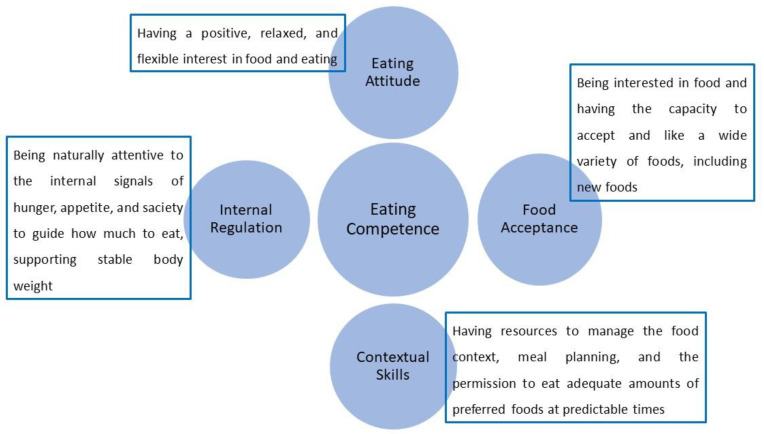
The four components of Eating Competence.

EC is the successful result of the four proposed components [9] described below and summarized in Figure 1. 

### 3.1. Eating Attitude

Eating attitude involves the beliefs, thoughts, and feelings that lead to behaviors that affect how individuals relate to food and influence food choices and, consequently, health [44]. The construction of eating attitude begins in childhood and is an aspect of great relevance for health promotion [45]. According to the concept of eating competence, the ideal eating attitude refers to being positive about food and eating, that is, enjoying eating and feeling comfortable about it [7], as well as having an interest in food, and showing self-confidence and tranquility concerning their food choices [2]. 

Excessive worries about food and eating, whether for aesthetic or health reasons, favor the development of distorted beliefs and feelings about food, leading to the continuous manifestation of a negative eating attitude [46]. In turn, these can lead to the search for an eating pattern out of the individuals’ reality [6,25,31,41]. In addition, individuals with negative attitude toward food and eating are more likely to demonstrate body dissatisfaction, as those who usually feel “very fat” or “very thin” or simply uncomfortable with their weight are more likely to feel ashamed of what they eat [7].

A New Zealand study on food habits and body weight (*n =* 294) found a significant link between the desire to lose weight and the belief in the association between chocolate cake and guilt. Participants who felt guilty about eating the cake had more difficulty maintaining or losing weight than those who associated the cake with celebration [47]. The association of chocolate cake with festivity was linked to better weight maintenance, consistent with the eating attitude proposed in the ecSatter model [1,6], emphasizing the relevance of eating attitude in food selection and health.

In 2006, a telephone survey with a representative sample of the American adult population (*n* = 2250) showed that individuals who consider themselves overweight report a lack of pleasure associated with eating compared to individuals who feel good about their body weight [48]. Six out of ten adults affirmed that they eat more quantity than they should. However, this type of statement was associated with behavioral aspects, being more present among individuals with higher scores on the stress assessment scale, among those who were overweight, who reported some concern with weight, or who were dieting to lose weight [48]. It is not possible to determine whether this judgment concerning overeating is a genuine result of high food consumption or whether it is a personal impression resulting from strict norms regarding body weight, food, and health. 

EC is associated with relevant behaviors in the context of nutritional health, such as greater satisfaction with body weight and a lower frequency of behaviors associated with eating disorders [9,19,20,25,49]. Body dissatisfaction seems to be a risk factor for overweight and eating disorders [20]. Queiroz et al. noticed that Brazilian adults who thought their body size was acceptable had higher EC scores than those who thought it was excessive (EC total score = 33.63 ± 7.56 vs. 27.7 ± 9.02; *p <* 0.000) [50]. Other studies on EC found a link between EC and body satisfaction. For example, among American college students (*n* = 1720), body mass index (BMI) was not as good a predictor of EC as weight satisfaction and desire to decrease weight [19]. Among low-income women, the lowest EC score is related to body weight dissatisfaction, a proclivity to overeat in reaction to external emotional stimuli, and eating disorder-related behaviors [9]. In a survey of 557 university students enrolling in an introductory nutrition course, researchers discovered that individuals who had never had an eating disorder had a higher average ecSI score than those who had a present or past eating disorder [49].

Eating-competent individuals usually tend to have less body dissatisfaction and less expression of weight control, as well as lower psychosocial characteristics related to disordered eating, fewer food dislikes, and greater food acceptance [20]. As EC increased, decreases were observed in the tendency towards bulimic thoughts, drive for thinness, and body dissatisfaction [20]. Regarding Eating Attitude, this component was inversely associated with restrained eating, body dissatisfaction, and desire to be thin [20]. Bulimic thoughts and feelings of uncontrolled hunger significantly increased as internal regulation decreased [20].

Individuals with positive eating attitude do not usually blame themselves for eating unhealthy foods [48]. In the psychological aspect, individuals with higher EC experience a positive and rewarding food context, so they feel able to eat what they like, according to their accessibility and sufficient quantity to meet their nutritional needs [2]. Furthermore, the higher the EC, the lower the food restrictions and the greater the acceptability of food [20], resulting in a more varied and healthy diet. 

Everything individuals think about certain foods and eating behaviors can influence their choices [51]. In this sense, it is important to better understand attitudes towards food and eating in the entire community, not just among people with eating disorders [44]. Considering that eating attitude influences food choices, understanding this component of EC seems to be relevant for nutrition diagnoses and health interventions.

### 3.2. Food Acceptance

The sensory characteristics of food, especially the taste, are identified determinants of food intake [24]. However, according to the EC concept, enjoyment and pleasure are important motivators for food selection [7].

Food choices are inserted in the priority order of human needs that can be understood from the perspective of Satter’s Hierarchy of Food Needs [52]. According to this proposal, the first basic need is to have enough food, which means food security from an economic and social standpoint. In this first level, individuals are driven by hunger and anxiety about getting enough to eat. The second need considers the subjective issue of acceptability, linked to food culture, social norms, and rules. In third comes the guarantee of having food availability for the next meals, indicating the possibility of planning the stock and budget for foods purchase. Following the hierarchy, the flavor comes fourth, after the first three basic needs are satisfied, and then the possibility of opening up new food experiences and eating unfamiliar foods. Finally, after all the above needs are met, the individual can consider instrumental reasons, such as searching for physical results (health and/or aesthetic) or cognitive and spiritual reasons [52]. At each level, needs must be satisfied before those at the next higher level can be experienced and addressed. The first three stages of the food need hierarchy are linked to issues involving the concept of Food and Nutritional Security [3].

Individually, the tendency to make food choices can be triggered by relatively simple stimuli, such as the content of sugar or fat in the food, the individual’s gastric capacity, the size of the portion presented, and even how much others eat [53]. However, external aspects affect those neurophysiological mechanisms, causing sensations such as pleasure and satiety, which are exacerbated or inhibited through logical reasoning about the consequences related to weight and health [53]. Moreover, concerning external issues, it is important to consider the various environments that influence food choices, as these environments make food available to the consumer [54]. In this sense, daily contemporary life is characterized by the abundance of attractive and energy-dense foods, which, combined with less need for physical activity, results in an “obesogenic” environment [53]. In many contemporary societies, some foods have become almost universally available and accessible, being purchased in many places, at any time, and by anyone [3,4,45]. The profusion of eating opportunities leads individuals to make numerous choices throughout the day, including the choice not to eat [1].

The acceptability of food is also related to the affective and symbolic value that the food represents, influencing the construction of preferences and aversions [3,45]. The taste construction and food preferences is a process that starts in early childhood; thus, the adequate development of this skill depends on the feeding practices from an early age. In this sense, among the feeding behaviors, it is recommended that the diet of the infant or young child be diversified, with repeated exposure to healthy foods and drinks, avoiding the offer of foods rich in salt, sugar, and flavor additives to provide the construction of healthy food preferences [55].

Food acceptance, in addition to being associated with economic, environmental, social, and cultural factors, is a determinant of food variety. It is known that dietary variety contributes to more complete and healthy nutrition, as no single food can provide all the nutrients that the human body needs [56]. A study of adults (4964 men and 4797 women) participating in the Continuing Survey of Food Intakes by Individuals demonstrated that dietary variety was strongly associated with better nutritional adequacy [57].

Food acceptance, according to the ecSatter model, highlights attitudes and behaviors such as: feeling calm in the presence of new and unfamiliar foods; feeling comfortable about food preferences (including foods with sugar, salt, fat, or other ingredients recognized as unhealthy); being able to make choices, accepting or refusing food offered, without constraints; having the ability to eat foods that you do not like very much if the situation demands it; and demonstrate curiosity about food, with an inclination to try new foods and, eventually, include new options in the food repertoire [2,7]. Thus, this component of EC seems to be an important aspect of eating behavior for health promotion.

### 3.3. Internal Regulation

This component is related to identifying the physical signs of hunger, appetite, and satiety, which will guide the amount of food to be eaten to contribute to the natural maintenance of healthy and stable body weight [7]. People with better internal regulation tend to have more regular meals, as they are naturally aware of the signs of hunger and can maintain a predictable rhythm of meals [8]. In addition, internal regulation allows confidence in the experience of satiety, which contributes not only to weight stability but also to satisfaction with the body shape [2]. Internal regulation is part of the central idea of intuitive eating and is also highlighted in mindful eating, as the improvement in awareness of internal and external experiences allows the individual to make more rational and less impulsive choices [6].

Internal regulation was linked to BMI, body size perception, and food consumption among Brazilian adults [2,7]. The absence of internal regulation is linked to the inability to recognize sensations related to hunger/satiety, as well as bulimic thoughts and feelings of uncontrollable hunger [20].

According to studies, a lack of EC is linked to bulimic thoughts, a feeling of uncontrollable eating, and a higher frequency of binge-eating episodes [9,20]. Generally, the more subtle bodily sensations happen automatically and, when such sensations are ignored, the signs of hunger and satiety end up being perceived later, when they are exacerbated [58]. This leads the individual to experience extremes of hunger and fullness. People who become used to external control over the amount they should eat—for example, restrained eaters and individuals who are dieting—may feel unable to trust their own ability to decide how much to eat [7]. Eating disorders can be triggered by dieting, but are not caused by it; however, it seems that self-imposed dietary restrictions are associated with lower levels of internal regulation [20].

According to the Satter proposal, to work on internal regulation, it is necessary to seek a balance between discipline and permission [2]. The discipline includes having regular meals in an appropriate environment, and permission involves the possibility of choosing the foods that will make up each meal, within the context limited by economic and social factors, but with the freedom to be able to eat the amount that satisfies hunger [13].

### 3.4. Contextual Skills

Food choices are situational and part of a process that requires multiple and interrelated decisions [1], e.g., a decision regarding what to eat is frequently tied to where to get it and how to prepare it, and a purchase decision might be linked to additional considerations including where to maintain food and how to deliver it [1]. In this sense, eating involves a series of actions and behaviors that include a variety of food handling steps, each of which requires distinct decision-making procedures, such as acquiring, preparing, and changing raw materials into meals [1].

In the ecSatter model, this component is linked to the ability to manage the food context, that is, develop food shopping strategies, plan meals, have cooking skills that enable food autonomy, and manage the time dedicated to preparing and consuming meals [2,20].

Food preparation skills have been positively associated with diet variety, increasing diet quality [59,60]. Cooking contributes to a healthier diet as it develops multiple knowledge about the different properties of food, making the cook acquire a conscious and objective attitude towards food, favoring the preparation of palatable, attractive, and good quality meals [53].

To minimize the replacement of fresh foods and regional culinary preparations by industrialized and ready-to-eat products, food systems should aim to preserve food cultures, encouraging the development of culinary skills to favor the consumption of artisanal and homemade meals [61]. The habit of cooking and preparing meals at home has shown a positive association with EC and healthy eating. Krall and Lohse, in a study to validate the ecSI with American women (*n* = 507; 18–45 y/o), found a positive relationship between EC and the habit of cooking at home. In addition, women classified as competent eaters (EC ≥ 32) reported that they like to cook and demonstrated more practical skills in managing their meals, including healthy aspects in food planning. As expected, women with greater contextual skills had lower BMI and presented a higher intake of FV [9]. Among Brazilian adults (*n* = 1810; 75% female), the contextual skills were positively associated with education level, age, BMI, food consumption, and income [62].

An American survey that interviewed 764 men and 946 women between the ages of 18 and 23 showed that those who reported more frequent preparation of their meals consumed amounts of fat, fruits, vegetables, whole grains, and calcium closer to dietary goals [63]. A recent Brazilian study showed that parents with autonomy and confidence in their cooking skills provide their children with a diet with fewer artificial and ultra-processed foods, indicating the importance of managing the food context in promoting the individual’s and families’ nutritional health [64]. Previous studies have revealed that parents with cooking practice behaviors play an important role in mediating their children’s consumption of FV [17,18].

According to Taylor, pleasure in eating is associated with pleasure in cooking [48]. To improve diet quality, interventions among young adults should invest in teaching practical and healthful food preparation skills [63]. Therefore, the development of food preparation skills using cooking classes has been considered a way to develop healthy eating and enable people to combine healthy habits with a wider variety of meals, resulting in increased independence in the implementation of healthy behaviors [6,65].

The ability to manage the food context proved to be especially important during the COVID-19 pandemic, where routines were entirely changed. A cross-sectional study performed in Brazil from 30 April to 31 May 2021 among a convenience sample of the Brazilian adult population (*n* = 302; 76.82% female) found that the measure of the contextual skills component decreased after the pandemic among those who gained weight (9.56 ± 3.43vs. 7.50 ± 3.95; *p* < 0.005); those who decreased the consumption of vegetables (9.78 ± 2.79 vs. 6.63 ± 3.40; *p* < 0.005), and those who increased the consumption of sugary beverages (9.19 ± 3.16 vs. 6.92 ± 3.70; *p* < 0.005) [66]. Moreover, individuals who used to buy ready-to-eat meals during the pandemic showed a reduction in total EC and all components (*p* < 0.005). On the other hand, there was no reduction in the contextual skill component among those who reported the habit to prepare their food [66].

The weakening of the transmission of cooking skills between generations favors the consumption of industrialized and unhealthy foods. Developing contextual skills allows the understanding that dealing with the food context is not a waste of time; on the contrary, it is an essential activity for life and for the promotion and maintenance of health, which can become a source of pleasure. Contextual skills are directly associated with the habit of planning meals, using all food groups and nutrition facts labels, as well as preparing meals, and eating at home more frequently [20].

## 4. Eating Competence Inventory

EC can be evaluated using the Satter Eating Competence Inventory (ecSI™2.0), a tool consisting of a 16-item self-administered questionnaire that assesses overall EC and its four components: eating attitude, composed of six items; food acceptance (three items); internal regulation (two items); and contextual skills (five items) [67].

Items are answered with the options: always; often; sometimes; rarely; and never. The score is obtained by the sum of the answers (always = 3; often = 2; sometimes = 1; rarely = 0; and never = 0), thus, the scores of the ecSI2.0™ can range from 0 to 48 [68]. The cutoff for the definition of eating competence is 32 and above [20,68]. The higher the ecSI2.0™ score, the higher the eating competence. There is no defined cutoff point for each of the four components [68]. However, in individualized approaches, to the extent that the score is deficient in one of the four components, it is possible to predict which skill the individual needs more attention and reinforcement.

The ecSI [69] was initially validated in 2007 with a sample of 832 US adult respondents (mean age 36.2 ± 13.4 years) without eating disorders, 78.7% female, white, educated, overweight, physically active, and food secure, providing support of content and construct validity, as well as internal consistency [20]. Its reliability was examined with 259 white females (26.9 ± 10.4 years), mostly food secure, with some college education, providing psychometric evidence about the reliability of the ecSI to measure EC but suggesting the revision of some items, as individuals with lower income tended to score lower on the ecSI [69]. In 2011, researchers revised the tool and made changes in the text of four items to favor the understanding of the content by individuals with lower income [9]. Construct validity of this instrument was demonstrated in a larger sample of 507 low-income women [9], aged 18 to 45 y/o, and results originated the ecSI/Low Income (ecSI/LI) [9]. The ecSI/LI was tested again in 2015 with 127 adults (35.8 ± 5.3 years) and proved to also be valid for higher-income groups [70]; this tool was named ecSI2.0™. A Confirmatory Factor Analysis was conducted by Godleski et al., to affirm factor structure resulting in the movement of one item from the Internal Regulation to the Eating Attitude subscale in the current version of the inventory (ecSI2.0™) [67].

Investigators and educators, under permission, can use the ecSI2.0™ to investigate the EC construct and track intervention outcomes with individuals of different levels of income and education [68]. Originally formulated in English, the ecSI2.0™ is translated into German, Arabic, Finnish, Japanese, Estonian, Spanish [71], and Brazilian-Portuguese [62]. Table 1 summarizes the main findings of the studies that measured EC using the ecSI in different population groups.

## 5. Eating Competence and Health

EC has been evaluated in several countries and is associated with health indicators, such as food consumption, maintenance of body weight, and the occurrence of diseases, and factors related to other health aspects, such as sleep quality, physical activity, stress management, and behaviors linked to eating disorders.

### 5.1. Eating Competence and Diet Quality

Despite not focusing on quantities or specific nutrients, the EC behavioral model is associated with diet quality [7]. Diet quality refers to the degree of adequacy of a dietary pattern compared to recommendations for healthy eating. Such recommendations are defined based on minimum parameters so that the diet provides all the necessary nutrients to promote and maintain health [56,78]. Among the dietary patterns explored in studies on EC, the consumption of FV is highlighted, following the recommendations for healthy eating [3,5]. The intake of FV is considered adequate when the usual rate is a minimum of five servings a day, totaling 400 g/day [79].

Positive associations between EC, food acceptability, and FV consumption have been described. For example, an American study, carried out with a convenience sample of 863 adults, compared the ecSI score with the responses of other instruments to investigate aspects of eating behavior, food acceptability, FV consumption, and sociodemographic data [20]. Among the instruments used, there were two related to food consumption and diet quality: *Food preference survey* (is an alternative to food frequency surveys, with a list of 62 food items, judged on a scale ranging from “dislike extremely” and “like extremely” with separate choices for “never tried” or “would not try”); and *Fruit and vegetable stage of change algorithm* (measures the stages of change—pre-contemplation, contemplation, preparation, action, and maintenance—for FV intake through responses that indicate the current intake and the intention to modify it). Regarding food consumption, individuals in the pre-contemplative stages of increased consumption of FV had lower scores on the ecSI than those who were already in the action and maintenance phases. Individuals with higher ecSI scores also had greater food acceptability and fewer dietary restrictions than those with lower EC [20]. The positive association between EC, food acceptability, and FV consumption was also confirmed in subsequent studies with low-income women [9,10,72]. EC also showed a positive association with diet quality in another study with American women (*n* = 149; age 18–50 y). Through a telephone interview, the researchers collected three days of 24 h dietary recall and the ecSI on the third day of the interview. The results showed that women classified as competent eaters (ecSI scores ≥ 32), had higher ingestions of fiber and vitamins A, E, C, and complex B, as well as magnesium, zinc, iron, and potassium. This study divided the group of women according to dietary patterns. The Prudent pattern, defined by the consumption of nutritious foods such as FV, and low-fat dairy products, was more prevalent among women classified as competent eaters. On the other hand, the Western pattern, associated with fatty, salty, and sugary foods, was observed more among women with lower scores on the ecSI [8,9].

A study in Brazil that looked at the relationship between EC and food intake and health outcomes among adults (*n* = 1810; 75% females) found that FV ingestion was strongly related to overall EC and its components [62]. The findings show that EC is linked to higher consumption of FV, which is related to improved health and protection against overweight [62].

Lohse et al. [12] investigated the relationship of EC with food consumption in 638 elderly at cardiovascular risk, participating in the Spanish clinical trial called *Prevención con Dieta Mediterranea* (PREDIMED). Comparing people with higher and lower EC, those with higher EC ingested more fruit and fish, consumed fewer dairy products, and consistently adhered to the Mediterranean diet [12]. In Finland, a recent cross-sectional study with 3147 adults (18 to 74 years old) at great risk for type 2 diabetes investigated whether EC is associated with lifestyle and metabolic risk factors for type 2 diabetes. The study showed that eating competent individuals (with a score ≥ 32 on the ecSI2.0™) had an improved diet quality, measured with an 18-item food intake validated questionnaire [76].

Among Brazilian adults (*n* = 1810) artificial juice (fruit-flavored soft drinks not made from fresh fruit) or soda consumption was found to be inversely related to EC [62]. Regular soda drinking is connected with a decreased intake of fruits and fiber, as well as a higher intake of junk foods and meals with a greater glycemic index [79]. Sugary drinks are also linked to increased energy consumption, a greater BMI, and a higher risk of medical complications [79].

EC is also associated with parental food-related actions that are favorable and mediate healthful food habits in young children, such as self-efficacy to serve FV and FV availability at home. In the USA, a study with parents of 4th-grade children (*n* = 339; 78% Hispanic), found that eating competent parents demonstrated more modeling, greater self-efficacy/outcome expectancies, greater in-home FV availability, and a higher frequency of eating breakfast and dinner with their children [17]. This research model was replicated in 2019 by Lohse et al. with a population of mostly white, non-Hispanic parents of 4th graders (*n* = 424; 94% white) and confirmed that the availability of FV continued to be greater in the homes of parents with higher EC, with results maintained even after adjustments for educational level [80].

Tylka et al. [18] looked at intuitive eating and EC as predictors of feeding practices (restriction, monitoring, pressure to eat, and dividing feeding responsibilities with their child), they found that mothers who allowed themselves unrestricted eating were less likely to inhibit their children’s food consumption; mothers who usually ate for physical (instead of sentimental) purposes and had contextual eating skills (e.g., mindful eating, planning regular and nutritious meals) were less likely to restrict their children’s food intake [18]. In the behavioral aspect, parents with higher EC had more dietary practices associated with the prevention of childhood obesity than parents classified as non-competent eaters [80].

In Finland, a study with adolescents that measured EC (using the ecSI translated into Finnish) found that EC was linked with a higher regularity of meals, a higher frequency of FV consumption, and more familiar healthy eating patterns [74].

### 5.2. Eating Competence and Risk Factors for Overweight and Non-Communicable Chronic Diseases (NCDs)

Overweight and obesity are important risk factors for developing NCDs, so the control and maintenance of an adequate weight have been recommended as a health goal. Lohse et al. [20] observed the relationship between EC and BMI in the ecSI validation study, with 863 healthy adults, aged between 18 and 71 years. On that occasion, individuals from the group of competent eaters reported a smaller lower percentage of BMI ≥ 25 compared to those from the group of non-competent eaters [20]. This association was also found in another study with low-income North American women, in which a lower ecSI score was related to a higher BMI [9]. A sampling of the adult population in Brazil yielded similar results (*n* = 11,810; 75% females) with high educational levels and high income, where eating competent individuals showed smaller BMI than non-eating competent ones [50].

The study with a sub-sample of 638 elderly participants at cardiovascular risk participating in the Spanish clinical trial *Prevención con Dieta Mediterránea* (PREDIMED) showed that individuals classified as competent eaters had lower BMI, higher High-density Lipoproteins (HDL), lower Low-density Lipoprotein (LDL), and lower fasting glucose rates, and participants with higher EC, despite reporting higher caloric intake, had lower BMI [12]. The association between EC and cardiovascular risk biomarkers was also documented in a smaller sample (*n* = 48), composed of men and women between 21 and 70 y/o, with dyslipidemia. Subjects classified as non-competent eaters had considerably higher levels of triglycerides and LDL compared to the competent eaters’ group [11]. In a Finnish study with participants in the StopDia (Stop Diabetes) survey, EC was linked to a lower rate of type 2 diabetes, visceral obesity, metabolic syndrome, hypertriglyceridemia, and greater insulin sensitivity [76]. These findings support the hypothesis that developing skills that increase EC may be a strategy to help control body weight, prevent metabolic syndrome and cardiovascular disease, and may help to prevent type 2 diabetes in the long term [76].

Subsequently, another Finnish study monitored 2291 individuals at high risk for type 2 diabetes to see if there were any links between changes in EC and changes in lifestyle, anthropometry, and glucose and lipid metabolism biomarkers [77]. During the intervention, participants were divided into three groups: (group i) received guidance to change their lifestyle through digital means; (group ii) received a lifestyle intervention based on group encounters, associated with digital guidance; and (group iii) control, who received written instructions about changing their diet and lifestyle. EC’s total score was 29.7 ± 7, with no difference in the score among the study groups. The EC total score increased among participants independent of the intervention type, being 0.4 in the digital (group i), 0.5 in the combined digital and group-based (group ii), and 0.7 in the control. Altogether, 40% of the participants were classified as competent eaters at the beginning, and 43% after one year (without differences among groups). Independent of initial EC, an improvement in EC was linked to enhanced HDL levels and a reduction in BMI and waist circumference. Among the components of EC, contextual skills, food acceptability, and eating attitude were associated with several of these changes, suggesting that EC may be a potential goal for lifestyle interventions to improve the health of people at risk for type 2 diabetes [78].

Regarding the management of body weight, recent findings show that in interventions for weight loss, the measurement of EC can be reduced at the beginning of treatment (up to four months), probably due to the concern with a restrictive diet. However, when the diet is accompanied by educational approaches, focusing on behavior change and increased physical activity, EC increases in the long-term (12 months) [75].

### 5.3. Eating Competence and Health-Related Aspects

Additional health-related aspects, including sleep quality and physical exercise, also show some association with EC. For example, individuals with higher EC tend to perceive themselves as being physically more active [14,20], and the relationship between low EC and low levels of physical activity is reported among low-income women [9].

Regarding sleep quality, a study with young university students found that overweight and obesity were linked to poor sleep quality and low EC, with results maintained after adjustments for the sociodemographic variables of the sample [73], suggesting that obesity prevention interventions for college students should include education components to improve EC and emphasize the importance of sleep quality [73]. Another research with university students evaluated the association between the number of sleep hours and EC, comparing students that slept eight hours or more per night with those who slept less than eight hours. The results show that those who slept less had even more poor eating habits, weaker internal food regulation, and more binge eating behaviors [15].

## 6. Conclusions

The rising prevalence of diseases linked to food and nutrition highlights the need to widen one’s perspective on food and its impact on health and well-being, considering not only nutrients and food combinations but also the behavioral dimensions of eating practices. Making food choices is a common and expected part of daily life, and it is an essential factor in everyone’s life [1]. Recent reports published by FAO and WHO [3,4] include healthy nutritional recommendations that take into account attitudes and behaviors that are in accordance with the behavioral model proposed by Satter [7]. In addition, researchers in the area of eating behavior emphasize the need to better understand attitudes toward food and eating in the general public by employing validated instruments to achieve this [44]. Considering that EC has been associated with diet quality and health outcomes, developing skills that increase EC might be a strategy to improve nutritional health and prevent obesity and other chronic diseases. The complexity of food choices has been examined on many fronts such as social, behavioral, and biological sciences, representing a great challenge for applying unique and simple theoretical models [1]. Multiple perspectives are suggested, as no single theory can clearly explain food decision making. Further studies are necessary to evaluate eating competence among different population groups and identify factors that might affect it, to stimulate policies and actions to improve EC among population groups.

## Figures and Tables

**Table 1:** Characterization of the studies that evaluated eating competence (EC) using the Ellyn Satter inventory (ecSI) in different populations.

Author/Year/Country	Number and Characterization of Participants	Study Objective	Main Findings
Lohse et al., 2007 [20] USA	832 (78.7% female; mean age 36.2 ± 13.4 years)	Examine ecSI psychometric properties and assess its validity to measure EC.	The ecSI is a valid tool to measure EC. Individuals with higher ecSI scores had better diet quality. Persons unsatisfied with weight had lower EC and showed higher feelings of cognitive restraint, disinhibition, hunger, weight management, and psychosocial characteristics related to disordered eating.
Stotts and Lohse, 2007 [69] USA	259 white females (food secure, with some college education; 26.9 ± 10.4 years)	Evaluate the ecSI reliability to measure EC.	The ecSI may be used to evaluate nutrition education designed to improve eating competence; however, some ecSI items may require revision.
Psota et al., 2007 [11] USA	48 (19 male and 29 female; 21–70 years old; LDL ≥ 110 mg/dL)	Explore the relationship between EC and the risk for cardiovascular disease.	Individuals with higher EC had lower cardiovascular risk. EC was positively correlated with HDL-cholesterol and inversely associated with blood pressure. Non-competent eaters showed higher cardiovascular risk.
Stotts Krall and Lohse, 2009 [72] USA	70 low-income adults in Pennsylvania (78.6% females; 31.7 ± 9.6 years)	Evaluate the congruence of the ecSatter model with the cognitive eating behaviors of a low-income audience.	EC was low for this low-income sample (mean 28.8 ± 8.3). For non-competent eaters, weight management plays an important role in meal planning and nutrition/health interests. They were more likely to express negative thoughts and feelings associated with eating, regardless of food security.
Stotts Krall and Lohse, 2010 [10] USA	25 low-income females (18–49 years old)	Examine the validity of EC self-report measures with low-income women.	Results provided a rationale for modifying the ecSI * and the development of the ecSI/LI for better use among low-income women.
Lohse et al., 2010 [12] SPAIN	638 elderly (62% women, ±67 years), at cardiovascular risk, recruited into the Spanish Trial PREDIMED.	Assess cross-sectional associations of EC with cardiovascular risk biomarkers and explore its relationships with the Mediterranean food pattern.	Individuals with higher EC ingested more fruit and fish, consumed fewer dairy products, and consistently adhered to the Mediterranean diet.
Clifford et al., 2010 [19] USA	1720 college students (67.9% female; 23.79 ± 7.8 years)	Determine whether BMI or attitudes about body weight were most predictive of EC in college students.	Students who were eating competent were more satisfied with their body weight, less likely to report the desire to lose weight, and had lower BMIs than students who were not eating competent. Weight satisfaction and desire to lose weight were better predictors of EC ** than BMI.
Krall and Lohse, 2011 [9] USA	507 low-income females (18 to 45 years)	Evaluate the construct validity of a version of the ecSI, as adapted for use in a low-income population.	Food acceptance, FV intake, food management, and self-reported physical activity were positively related to ecSI/LI scores. BMI, dissatisfaction with body weight, tendency to overeat, and disordered eating were negatively associated with ecSI/LI scores.
Lohse et al., 2012 [8] USA	149 women (56% white, 64% food secure, 86% 18–50 y)	Determine if EC is associated with dietary intake and diet patterns among low-income women.	Competent eaters (EC ≥ 32) showed better diet quality. The healthful dietary pattern showed a positive relationship with EC. The pattern characterized by foods higher in fat, salt, and sugar, was inversely related to EC.
Lohse and Cunningham-Sabo, 2012 [17] USA	339 Parents (37.2 ± 7.7 years; 78% Hispanic; 89% female) of 4th graders.	Determine if EC was a moderator of parents FV-related eating behaviors that mediate healthful eating in 4th-grade children.	Eating-competent parents demonstrated more modeling behaviors related to food preparation and fruits/vegetables; greater self-efficacy/outcome expectancies and greater in-home fruit/vegetable availability. Measuring EC may contribute to understanding parent behavior as a mediator in school-based nutrition interventions.
Lohse et al., 2013 [14] USA	512 low-income women (30.7 ± 7.5 years)	Compare EC between women who perceive being physically active with those who do not, and examine their responses to an online physical activity program.	EC was higher for physically active women. The perception of being physically active was higher in eating competent low-income women.
Brown et al., 2013 [49] USA	557 students (67.6% females; 343 ages 18–20 years; 180 ages 21–26 years)	Describe EC in students enrolled in an introductory nutrition course.	A total of 47.4% were classified as eating competent. Mean EC scores were higher for males than females and for students who never had an eating disorder.
Tylka et al., 2013 [18] USA	180 mothers of 2- to 5-year-old children	Identify the adaptive maternal eating behaviors that contribute incrementally to their child feeding practices.	Mothers who had greater EC and engaged in intuitive eating reported better feeding practices. Interventions to develop EC and skills to eat intuitively may favor positive feeding environments for children.
Quick et al., 2014 [73] USA	1252 college students (59% female; 18–24 years; 80% white)	Examine the relationships between sleep, eating, and exercise behaviors; work time pressures; and weight of young adults.	Lower EC was significantly associated with overweight/obesity.
Lohse et al., 2015 [13] USA	288 low-income women (30.7 ± 7.8 years)	Produce and evaluate an online program for low-income women following the ecSatter model.	ecSI2.0 demonstrated good internal consistency. Only 39% of the sample was categorized as competent eaters. After About Eating, women increased their food-management skills, confirming the usefulness of this online program.
Lohse, 2015 [70] USA	127 parents (35.8 ± 5.3 years) of preschool-age children; 75.5% not considered low-income.	Determine if the ecSI/LI could be applied to a general audience.	Eating competence can be accurately measured with the ecSI2.0 (formerly called the ecSI/LI), regardless of income.
Quick et al., 2015 [16] USA	1035 college students (61% female; 18–24 years)	To explore the associations of EC with sleep behavior and quality.	Competent eaters (EC ≥ 32) are more likely to have better overall sleep quality and fewer sleep-related problems.
Tilles-Tirkkonen et al., 2015 [74] FINLAND	976 Finnish adolescents (10–17 years old)	Explore a Finnish translation of the ecSI2.0 for evaluating EC and its association with food selection, meal patterns, and related psychobehavioral factors.	EC was associated with diet quality and more health-promoting family eating patterns. Competent eaters more often perceived their body size as appropriate and had less often tried to lose weight.
Quick et al., 2016 [15] USA	1252 college students (59% female; 18–24 years old)	To describe sleep behaviors and examine associations of sleep duration with eating and physical activity.	Those who slept < 8 h/night had significantly more negative eating attitudes, poorer internal regulation of food, and greater binge eating scores.
Lohse et al., 2018 [75] USA	101 women (premenopausal, mostly college-educated, body mass index >25)	To examine changes in EC in a 12-month weight-loss intervention.	Weight loss interventions that introduce concerns about eating attitudes, behaviors, and foods can reduce EC. Extending the measurement range is more appropriate as it allows sufficient time for the individual to acquire self-efficacy, better reflecting the intervention’s impact on EC.
Godleski et al., 2019 [67] USA	2010 from demographically heterogeneous samples (mean age in the thirties; 89% female; 74% white; 18% Hispanic)	Examine the structural validity of the ecSI 2.0.	Findings supported retaining all 16 items and migration of 1 item from the Internal Regulation to Eating Attitudes subscales. The psychometric integrity of the 16-item ecSI 2.0 was affirmed.
Tilles-Tirkkonen et al., 2020 [76] FINLAND	3147 Finnish adults (18–74 years) at an increased risk for type 2 diabetes, participated in the StopDia study.	Investigate whether EC is associated with diet or risk factors and the prevalence of type 2 diabetes in individuals with type 2 diabetes risk.	EC was associated with diet quality and lower prevalence of previously undiagnosed type 2 diabetes, abdominal obesity, metabolic syndrome, hypertriglyceridemia, and better insulin sensitivity. Enhancing EC could support the prevention of type 2 diabetes.
Queiroz et al., 2020 [62] BRAZIL	662 Brazilian adults (74.9% female, 40.33 ± 12.55 years, high schooling and income)	Translate and validate the ecSI2.0 from English to Brazilian-Portuguese.	The ecSI2.0BR is a useful tool designed to measure EC in the Brazilian population, showing good reproducibility and internal consistency.
Queiroz et al., 2020 [50] BRAZIL	1810 Brazilian adults (75% females, mostly up to 40 years old, with a high education level and income)	Associate EC with food consumption and health outcomes in the Brazilian adult population.	EC was inversely associated with BMI. EC did not differ among males and females, and respondents up to 40 years old presented a lower total score. Individuals with adequate consumption of FV presented the best scores for total EC.
Queiroz et al., 2021 [66] BRAZIL	302 Brazilian adults (76.82% females)	Compare EC among Brazilian adults before and during the coronavirus pandemic.	EC total score lowered during the pandemic and the decrease was worst among individuals who reported weight gain, decreased FV consumption, and an increase in sugar consumption.
Aittola et al., 2021 [77] FINLAND	2291 adults at increased risk of type 2 diabetes.	Investigate the associations of changes in EC with changes in lifestyle, anthropometrics and biomarkers of glucose and lipid metabolism.	EC was associated with an increase in diet quality, high-density lipoprotein cholesterol, and with decreased BMI and waist circumference. EC could be a potential target in lifestyle interventions to improve the cardiometabolic health of people at type 2 diabetes risk.

* ecSI: eating competence Satter Inventory; ** EC: eating competence; ecSI/LI: eating competence Satter Inventory for Low Income audience; ecSI2.0: the new version of eating competence Satter Inventory; ecSI2.0/BR: the Brazilian version of the eating competence Satter Inventory; FV: fruits and vegetable; and BMI: Body Mass Index.

## Data Availability

Not applicable.
